# Fault mechanism analysis and diagnosis for closed-loop drive system of industrial robot based on nonlinear spectrum

**DOI:** 10.1038/s41598-022-21691-6

**Published:** 2022-11-01

**Authors:** Lerui Chen, Shengjun Wen, Haiquan Wang, Heyu Hu

**Affiliations:** 1 Zhongyuan University of Technology, Zhongyuan Road NO.41 of Zhengzhou, Zhengzhou, 450007 China; 2 State Key Laboratory for Manufacturing Systems Engineering, Xi’an JiaoTong University, Xi’an, 710049 China

**Keywords:** Electrical and electronic engineering, Mechanical engineering

## Abstract

To solve the problem of nonlinear characteristics neglecting and fault mechanism analysis lacking in fault diagnosis research, a new method of fault mechanism analysis and diagnosis based on nonlinear spectrum is proposed. Firstly, based on the Permanent Magnet Synchronous Motor (PMSM) model of robot, the first 4-order spectrums based on nonlinear output frequency response function (NOFRF) in different states are obtained by batch calculation method. Secondly, the high-frequency spectrum distribution rule of NOFRF spectrum in different states are analyzed. Finally, in the closed-loop simulation environment of robot, the identification method based on data-driven is adopted for NOFRF spectrum calculation to verify power loss fault of PMSM. Meanwhile, the fault diagnosis experiment is also carried out. The experimental results indicate that the key characteristics distribution rule of NOFRF spectrums in the real environment is consistent with the theoretical analysis results, and compared with the traditional fault feature extraction methods by output signal, the diagnosis with fault feature of NOFRF spectrum for industrial robot closed-loop drive system has the highest accuracy, which verifies the validity of NOFRF spectrum as the fault feature.

## Introduction

Industrial robot plays an important role in the intelligent manufacturing. When the fault occurs, it will not only affect the quality of products, but also increase the cost of equipment maintenance. For the industrial robot, permanent magnet synchronous motor (PMSM) is often adopted as the power source^1,2^. Because of the continuous load operation, the fault probability of PMSM is high among all possible faults of robot^3^. In order to eliminate the threat of PMSM fault to the safe operation of industrial robot, it is very important to realize fast and accurate diagnosis of the system^4^, whereas, the fault mechanism analysis is the premise of fault accurate diagnosis.

Fault mechanism analysis is to analyze the mapping relationship among system fault symptoms, fault characteristic parameters and fault reasons. Through the analysis of fault mechanism, the selection of fault characteristic parameters is more targeted and scientific, and it is also the key to improve the fault diagnosis accuracy. Fault diagnosis by traditional methods mainly deal with the equipment temperature, noise, current, voltage and vibration signal by experience or device to find out fault reasons of system. For example, in Refs.^5–7^, the vibration signals of system under different faults were collected, and then the signal processing methods were adopted to analyze the variation rule from vibration signals, finally, the relevant classifiers were designed to carry out the diagnosis of system. Ref.^8^ proposed a current detection method for motor traction system, by comparing the synchronous currents of estimated and measured, the diagnosis of motor can be achieved. Ref.^9^ utilized a wavelet packet transform to extract the fault feature from current signal of permanent magnet synchronous motor (PMSM), and the classifier was designed to realize the fault diagnosis of PMSM. These diagnosis methods above lack the fault mechanism analysis, so the selection of fault signal is subjective and blind, which is not conducive to improve the accuracy of fault diagnosis. Up to now, there are many researches on PMSM fault diagnosis, most of them are based on signal processing. In Ref.^10^, PMSM was excited by low sinusoidal voltage, and the fault of stator inter-turn short circuit was estimated by detecting and analyzing the harmonic components of current signal in windings. In Refs.^11,12^,the PMSM stator current in time domain was collected and then converted into frequency domain by Fast Fourier transform (FFT) for fault analysis. In Ref.^13^, the empirical mode decomposition (EMD) was performed for PMSM stator winding current, and the instantaneous frequency component in current was extracted as the permanent magnet leakage fault feature for diagnosis. In Ref.^14^, the continuous wavelet transform (CWT) was adopted to transform the PMSM stator current signal in time domain into time-frequency domain signal to realize the fault diagnosis of magnetic flux leakage. These researches above extracted the fault features from PMSM fault signal in the form of time domain, frequency domain or time-frequency domain, and then classifiers were designed to achieve the fault diagnosis. However, there are at least two defects of such researches. Firstly, the selection of fault signal was blind, lacking the analysis of characteristic change rule in different states, which cannot reflect the mapping relationship between fault type and fault characteristic parameters. Secondly, the fault signal was selected based on single output of the system, completely ignoring the influence of system nonlinear characteristics on the mapping relationship between fault characteristics and fault reasons, which may lead to the unobvious fault characteristics between different states. Therefore, it is necessary to analyze the fault mechanism in combination with the nonlinearity of system. However, the research of this area is still a blank at present.

The analysis method by nonlinear spectrum is to transform the Volterra kernel in time domain into frequency domain by multi-dimensional Fourier transformation, which can obtain the nonlinear spectrum transfer characteristics of the system. Theoretical and experimental results indicate that when the system state changes, the nonlinear spectrum characteristics will also change^15,16^.Therefore, the nonlinear spectrum characteristics can be adopted to analyze the fault mechanism of complex system. Up to now there are two models of nonlinear spectrum: generalized frequency response function (GFRF) and nonlinear output frequency response function (NOFRF). GFRF is an extension of linear system frequency response function, but the structure of GFRF is complex and the calculation is large^17–19^. NOFRF is a one-dimensional spectrum, which can be seen as the projection of multi-dimensional GFRF function on the hyperplane. The model of NOFRF is simple and the calculation quantity is small, which is widely used in the field of system nonlinear spectrum research^20–23^.

In this paper, NOFRF spectrum is adopted to analyze the fault mechanism and achieve the diagnosis of PMSM power loss. Firstly, the common fault reasons of PMSM power loss are found, then on the basis of PMSM mathematical model, the first 4-order NOFRF spectrums of different fault reasons are obtained by batch identification algorithm, and the change rules of key NOFRF spectrum characteristics under different states are analyzed. Finally, an industrial robot simulation experiment platform is established, and the first 4-order NOFRF spectrums of robot closed-loop drive system are obtained by data-driven method. The rest of this paper is organized as follows. Section Methodology presents the theory of NOFRF spectrum. In Section Fault mechanism analysis of PMSM based on NOFRF spectrum, the fault mechanism of open-loop PMSM system is analyzed by NOFRF spectrum, and the comparative experiments are carried out. Section Experimental verification and discussions verifies the theoretical analysis of NOFRF spectrum in closed-loop robot operation environment. Conclusions can be drawn in Section Conclusions.

## Methodology

### Nonlinear spectrums based on NOFRF

For a continuous time invariant nonlinear system, the output can be expressed as Eq. (1)^18^.

1 $$y_{n} (t) = \int_{ - \infty }^{\infty } {\int_{ - \infty }^{\infty } { \cdots \int_{ - \infty }^{\infty } {h_{n} (\tau_{1} ,\tau_{2} , \cdots \tau_{n} )\prod\limits_{i = 1}^{n} {u(t - \tau_{i} )d\tau_{i} } } } } ,$$

where, $u(t)$ and $y_{n} (t)$ are input and the *n*-th output of system, respectively. $h_{n} (\tau_{1} ,\tau_{2} , \cdots \tau_{n} )$ is the *n*-th Volterra kernel, and the frequency domain of $h_{n} (\tau_{1} ,\tau_{2} , \cdots \tau_{n} )$ can be shown as Eq. (2).

2 $$H_{n} ({\text{j}}\omega_{1} ,{\text{j}}\omega_{2} , \cdots ,{\text{j}}\omega_{n} ) = \, \int_{ - \infty }^{\infty } { \cdots \int_{ - \infty }^{\infty } {h_{n} (\tau_{1} ,\tau_{2} , \cdots \tau_{n} )} } e^{{ - {\text{j}}(\omega_{1} \tau_{1} + \omega_{2} \tau_{2} + \cdots + \omega_{n} \tau_{n} )}} \prod\limits_{i = 1}^{n} {d\tau_{i} } .$$

**Figure 1:**
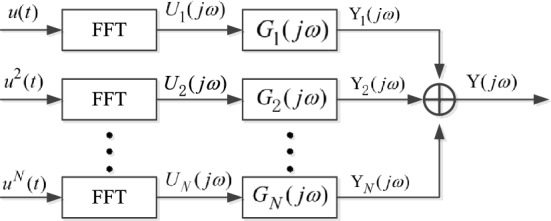
Output frequency response of nonlinear systems based on NOFRF.

In Eq. (2), $H_{n} ({\text{j}}\omega_{1} ,{\text{j}}\omega_{2} , \cdots ,{\text{j}}\omega_{n} )$ is called generalized frequency response function (GFRF), which can describe the system nonlinear characteristics, but it contains a large amount of data.To simplify the calculation, nonlinear output frequency response function (NOFRF) was proposed in Ref.^20^, which is shown as Eq. (3). It is one-dimensional function with small amount of data, which can reduce the amount of calculation.

3 $$G_{n} ({\text{j}}\omega ) = \frac{{\int_{{\omega_{1} + \omega_{2} + \cdots + \omega_{n} = \omega }} {H_{n} ({\text{j}}\omega_{1} ,{\text{j}}\omega_{2} , \cdots ,{\text{j}}\omega_{n} )\prod\limits_{i = 1}^{n} {U({\text{j}}\omega_{i} )d({\text{j}}\omega_{i} )} } }}{{\int_{{\omega_{1} + \omega_{2} + \cdots + \omega_{n} = \omega }} {\prod\limits_{i = 1}^{n} {U({\text{j}}\omega_{i} )d({\text{j}}\omega_{i} )} } }},$$

where, $\int_{{\omega_{1} + \omega_{2} + \cdots + \omega_{n} = \omega }} {H_{n} ({\text{j}}\omega_{1} ,{\text{j}}\omega_{2} , \cdots ,{\text{j}}\omega_{n} )\prod\limits_{i = 1}^{n} {U({\text{j}}\omega_{i} )d({\text{j}}\omega_{i} )} }$ is the integral of $H_{n} ({\text{j}}\omega_{1} ,{\text{j}}\omega_{2} , \cdots ,{\text{j}}\omega_{n} )\prod\limits_{i = 1}^{n} {U({\text{j}}\omega_{i} )}$ on hyperplane $\omega_{1} + \omega_{2} + \cdots + \omega_{n} = \omega$.$U({\text{j}}\omega_{i} )$ is the Fourier transformation of input $u(t)$, and $\int_{{\omega_{1} + \omega_{2} + \cdots + \omega_{n} = \omega }} {\prod\limits_{i = 1}^{n} {U({\text{j}}\omega_{i} )d({\text{j}}\omega_{i} )} } \ne 0$. Therefore, the output frequency response of nonlinear system can be represented as Fig. 1.

By introducing the concept of NOFRF, the output spectrum of nonlinear systems can be expressed as Eq. (4).

4 $$Y({\text{j}}\omega ) = \sum\limits_{n = 1}^{N} {Y_{n} ({\text{j}}\omega ) = } \sum\limits_{n = 1}^{N} {G_{n} ({\text{j}}\omega )} U_{n} ({\text{j}}\omega ),$$

where, $Y_{n} ({\text{j}}\omega )$ is the *n*-order output in frequency-domain. $U_{n} ({\text{j}}\omega )$ is defined as Eq. (5).

5 $$U_{{\text{n}}} (j\omega ) = \frac{{{\raise0.7ex\hbox{$1$} \!\mathord{\left/ {\vphantom {1 {\sqrt n }}}\right.\kern-\nulldelimiterspace} \!\lower0.7ex\hbox{${\sqrt n }$}}}}{{(2\pi )^{n - 1} }}\int_{{\omega_{1} + ... + \omega_{{\text{n}}} = \pi }} {\prod\limits_{{{\text{i}} = 1}}^{{\text{n}}} {U(j\omega_{{\text{i}}} )d(j\omega_{{\text{i}}} )} } .$$

Compared with the generalized frequency response function (GFRF) as Eq. (2), NOFRF can avoid the dimension disaster. Moreover, studies have been demonstrated that NOFRF can explain a wide range of nonlinear phenomena^21,22^, such as high-harmonic phenomenon.

### The calculation of NOFRF spectrum

The purpose of fault mechanism analysis using nonlinear spectrum is to obtain the nonlinear spectrum change rules of the system in different states. Therefore, how to calculate the NOFRF spectrum is important. At present, there are two calculation methods: batch algorithm and identification algorithm. According to the batch algorithm in Ref.^24^, using input signal $A_{i} u^{*} (t)$ to excite M times, where $i = 1,...,{\text{M}},{\text{M}} \ge {\text{N}},A_{i}$ is constant, and $A_{{\text{M}}} > A_{{\text{M - 1}}} > ... > A_{1} > 0$.$u^{*} (t)$ is time-vary signal, whose frequency-domain form is $U^{*} (j\omega )$. The system output in frequency-domain is $Y_{i} (j\omega )$,$i = 1,...,{\text{M}}$,thus,

6 $$Y_{i} (j\omega ) = B_{i}^{*} (j\omega )G^{*} (j\omega ),$$

where,$$Y_{i} (j\omega ) = [Y_{1} (j\omega )...Y_{M} (j\omega )]^{T}$$. $$B_{i}^{*} (j\omega ) = \left[ \begin{gathered} A_{1} U_{1}^{*} (j\omega ) \cdots A_{1}^{N} U_{N}^{*} (j\omega ) \hfill \\ \, \vdots \, \vdots \hfill \\ A_{M} U_{1}^{*} (j\omega ) \cdots A_{M}^{N} U_{N}^{*} (j\omega ) \hfill \\ \end{gathered} \right]$$.

$G^{*} (j\omega ) = [G_{1}^{*} (j\omega )...G_{N}^{*} (j\omega )]^{T}$ is the NOFRF spectrum, which can be obtained by Eq. (7).

7 $$G^{*} (j\omega ){ = [(}B_{i}^{*} (j\omega ))^{T} B_{i}^{*} (j\omega )]^{ - 1} {(}B_{i}^{*} (j\omega ))^{T} Y_{i} (j\omega ).$$

**Figure 2:**
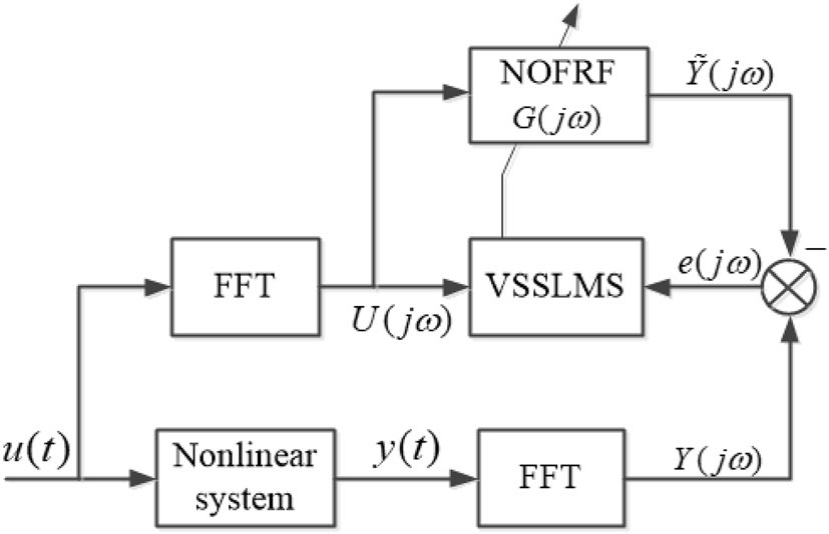
The schematics of NOFRF identification by VSSLMS.

The accuracy of NOFRF spectrum obtained by batch algorithm is high, but it will cost a lot of time due to the existence of inverse calculation in Eq. (7). The identification algorithm is a black box operation based on data-driven, and the calculation of NOFRF spectrum can be realized only by input and output of the system, which can avoid the complex inversion calculation. Therefore, some scholars try to adopt identification algorithm to calculate NOFRF spectrum. Ref.^25^ proposed the least mean square(LMS) adaptive method for NOFRF online identification of hydro generator. Ref.^26^ proposed block least mean square(BLMS) algorithm to identify NOFRF in circuit system. In order to improve the performance of identification accuracy and speed, based on the existing least mean square (LMS) algorithm, this paper proposed a novel identification algorithm of variable step size least mean square (VSSLMS) to calculate the NOFRF spectrum of PMSM. The schematics of VSSLMS can be shown as Fig. 2.

In Fig. 2, $u(t)$ is the input of system. $y(t)$ is the output of system. $Y(j\omega )$ is the actual spectrum of system output. $\tilde{Y}(j\omega )$ is the estimated spectrum of system output. $e(j\omega )$ is the error between $Y(j\omega )$ and $\tilde{Y}(j\omega )$.$U(j\omega ) = [U_{1} (j\omega ),U_{2} (j\omega ), \cdots ,U_{k} (j\omega ), \cdots ,U_{N} (j\omega )]^{T}$,$G(j\omega ) = [G_{1} (j\omega ),G_{2} (j\omega ), \cdots ,G_{k} (j\omega ), \cdots ,G_{N} (j\omega )]^{T}$. It can be seen from Fig. 2 that the essence of VSSLMS algorithm is an optimization problem with the goal of $e(j\omega )$ minimization. Through continuous iteration and updating, when $e(j\omega )$ reaches the minimum value, all parameters reach the optimal value. At this moment, the optimal parameters are extracted and $G(j\omega )$ can be solved.

The identification model of VSSLMS can be write as Eq. (8).

8 $$G_{{k{ + 1}}} (j\omega ){ = }G_{k} (j\omega ){ + }\mu_{k} \frac{{e_{k} (j\omega )U_{k}^{T} (j\omega )}}{{\lambda + U_{k}^{T} (j\omega )U_{k}^{ * } (j\omega )}},$$

where, $e_{k} (j\omega )$ is the error between $\tilde{Y}_{k} (j\omega )$ and $Y_{k} (j\omega )$, $U_{k}^{T} (j\omega )$ is the input transpose in frequency domain, $\lambda$ is a constant, ‘$*$’denote the complex conjugate, $\mu_{k}$ is the step size, which can be expressed as Eq. (9).

9 $$\mu_{{\text{k + 1}}} = \left\{ \begin{gathered} \mu_{{{\text{max}}}} , \, \mu_{{\text{k}}} \ge \mu_{{{\text{max}}}} \hfill \\ \mu_{{{\text{min}}}} , \, \mu_{{\text{k}}} \le \mu_{{{\text{min}}}} \hfill \\ \mu_{{\text{k}}} ,{\text{ other}} \hfill \\ \end{gathered} \right.,$$

where, $\mu_{{\text{k + 1}}} = \alpha \mu_{{\text{k}}} + \beta \left| {e_{{\text{k}}} (j\omega )} \right|^{2} ,0 < \alpha < 1,\beta > 0$. $\mu_{{{\text{max}}}}$ is the upper limit of $\mu_{{\text{k}}}$.$\mu_{{{\text{min}}}}$ is the lower limit of $\mu_{{\text{k}}}$.

In Eq. (9), $\mu_{{\text{k}}}$ is affected by error $e_{{\text{k}}} (j\omega )$, that’s to say, when $e_{{\text{k}}} (j\omega )$ is large, $\mu_{{\text{k}}}$ is also large, which will accelerate the convergence speed of algorithm. When $e_{{\text{k}}} (j\omega )$ is small, $\mu_{{\text{k}}}$ is also small, which will reduce the steady-state error of the algorithm. From the analysis above, it can be found that the adaptive identification algorithm as proposed can take into account both convergence speed and estimation error. The specific steps of VSSLMS algorithm are as follows.

- **Step 1**: Collecting the input $u{(}t{)}$ and output $y{(}t{)}$ of system, and then transforming them by FFT, which can obtain $U{(}j\omega {)}$ and $Y{(}j\omega {)}$, respectively.
- **Step 2:** Calculating the estimated output $\tilde{Y}{(}j\omega {)}$ by Eqs. (4) , (8).
- **Step3:** Calculating the error $e_{k} {(}j\omega {)}{ = }\tilde{Y}{(}j\omega {)} - Y{(}j\omega {)}$. if $\left| {e_{k} {(}j\omega {)}} \right|\varepsilon$,ending the identification. Otherwise, jumping to sstep 4.
- **Step 4:** Updating the step size $\mu_{k + 1}$, and then calculating the NOFRF vector $G_{{k{ + 1}}} ({\text{j}}\omega )$.
- **Step 5:** Making $k{ = }k{ + 1}$, and then jumping to step 2.

## Fault mechanism analysis of PMSM based on NOFRF spectrum

### Data acquisition

In the process of continuous operation of industrial robot, power loss fault may be occurred, which is mainly caused by the following reasons: stator winding in-turn short circuit, permanent magnet leakage flux or the two reasons exist at the same time. Therefore, this paper analyzes the fault mechanism of PMSM adopted in the driving system of industrial robot. According to Ref.^27^, the mathematical model of PMSM can be expressed as follows.

10 $$i_{d}^{\prime } + \frac{{\text{r}}}{{\text{L}}}i_{d} - \frac{{2{\text{J}}}}{{3\phi_{{\text{g}}} }}\omega_{r} \omega_{r}^{\prime } - \frac{{2{\text{B}}}}{{3\phi_{{\text{g}}} }}\omega_{r}^{2} - \frac{{2{\text{T}}_{l} }}{{3\phi_{{\text{g}}} }}\omega_{r} = 0,$$

where, $i_{d}$ is armature currents of stator winding on *d*-axis. ${L}$ is stator winding inductance. $\phi_{{\text{g}}}$ is flux linkage produced by permanent magnet. ${r}$ is stator winding resistance. ${J}$ is rotor moment of inertia. ${T}_{{l}}$ is load torque. ${B}$ is rotor damping coefficient. $\omega_{r}$ is rotor angular velocity. $i_{d}$ is input and $\omega_{r}$ is output of the system. Stator winding inter-turn short circuit and permanent magnetic leakage flux affect parameters ${r}$、$\phi_{{\text{g}}}$ in Eq. (10), respectively. In order to explain the influence of different fault reasons on NOFRF spectrum, this paper chooses normal state, two single fault reasons and one composite fault reason for analysis, which is shown as Table 1.

**Table 1:** The fault reasons of PMSM.

State	Label	Fault reason
Normal	R0	None
Power loss	R1	Stator winding inter-turn short circuit
	R2	Permanent magnetic leakage flux
	R3	Stator winding inter-turn short circuit and permanent magnetic leakage flux

### The influence of different fault reasons on NOFRF spectrum

At present, most of fault diagnosis researches adopted signal in frequency domain^28,29^ or time-frequency domain^30–32^ for analysis. In order to illustrate the effectiveness of NOFRF spectrum to be as the fault feature for fault mechanism analysis, this paper adopts frequency domain by Fourier transform and time-frequency domain by short-time Fourier transform (STFT) as comparative experiments.

#### Frequency domain analysis

**Figure 3:**
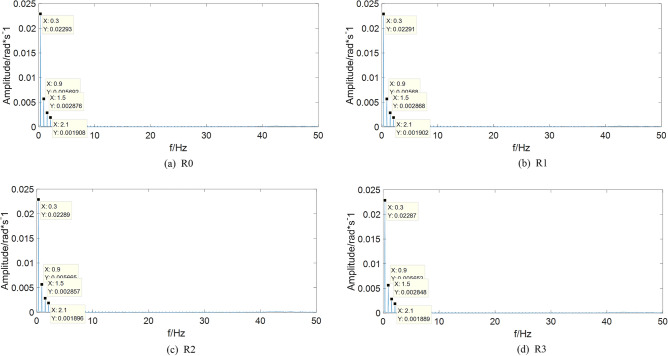
Frequency domain of output signal in different states.

The frequency domain analysis method by Fourier transform is a common signal processing method. Making the input signal as $i_{d} {(t) = 10cos(0}{.6\pi t)}$, collecting the motor angular velocity speed signal $\omega_{r}$, and then the frequency domain spectrum of different states are obtained by Fourier transform (FT), which can be shown as Fig. 3.

From Fig. 3, it can be seen that both the fundamental frequency spectrum (0.3 Hz) and the high-frequency harmonics, such as three multiple-frequency, five multiple-frequency and seven multiple-frequency, appear in the FT spectrum. With the frequency increasing, the high-frequency harmonics show an attenuation trend, which fully verifies the fact that the system has nonlinear characteristics. However, after the FT spectrum of different states being compared and analyzed, it is found that the FT spectrum between the normal state and fault state are different, but the difference is not very obvious. For example, the amplitudes of three multiple-frequency harmonic in normal state, fault state caused by stator winding inter-turn short circuit, fault state caused by permanent magnetic leakage flux, fault state caused by stator winding inter-turn short circuit and permanent magnetic leakage flux are 0.005692, 0.005680, 0.005665 and 0.005652, respectively. So, it can be draw a conclusion that the frequency domain spectrum is not sensitive to fault. If the frequency domain spectrum is taken as the fault feature parameter of PMSM for diagnosis, the feature information of different states may overlap, which will affect the accuracy of classifier recognition.

#### Time-frequency domain analysis

**Figure 4:**
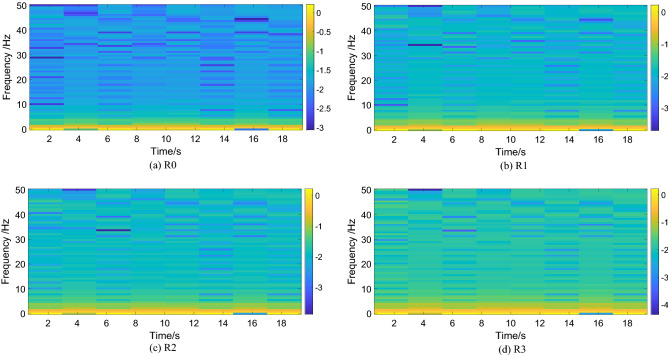
STFT spectrum of output signal in different states.

Short-time Fourier Transform (STFT) is a common method for non-stationary signal analysis, which combines both time and frequency characteristics. Compared with Fourier transform (FT), STFT contains more information. In this paper 128 size Hamming window is adopted in STFT, and the specific STFT spectrum of motor angular velocity speed $\omega_{r}$ in different states are shown as Fig. 4.

As can be seen from Fig. 4, the difference of STFT spectrum between normal state and fault state is obvious. In normal state, the frequency components distribution of STFT spectrum are relatively concentrated, while the frequency components distribution of STFT spectrum in fault state are divergent, which shows that the time-frequency distribution obtained by STFT has a good frequency resolution, which can effectively distinguish the normal state and the fault states. However, the difference of STFT spectrum between fault states caused by different reasons is unobvious. So, the distinction of fault characteristics is also not obvious. If it is adopted as fault feature for diagnosis, different faults cannot be distinguished effectively.

#### NOFRF spectrum analysis

**Figure 5:**
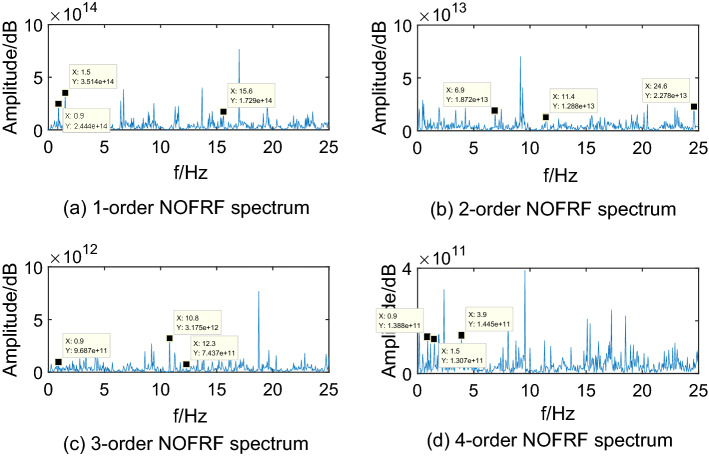
NOFRF spectrum of first 4-order in R0.

**Figure 6:**
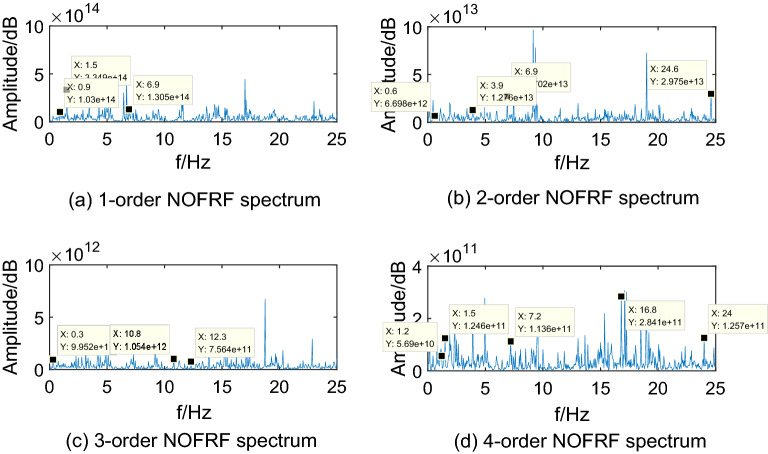
NOFRF spectrum of first 4-order in R1.

**Figure 7:**
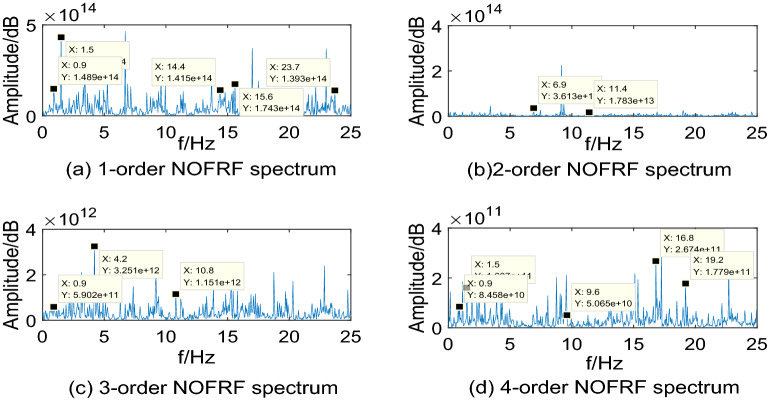
NOFRF spectrum of first 4-order in R2.

**Figure 8:**
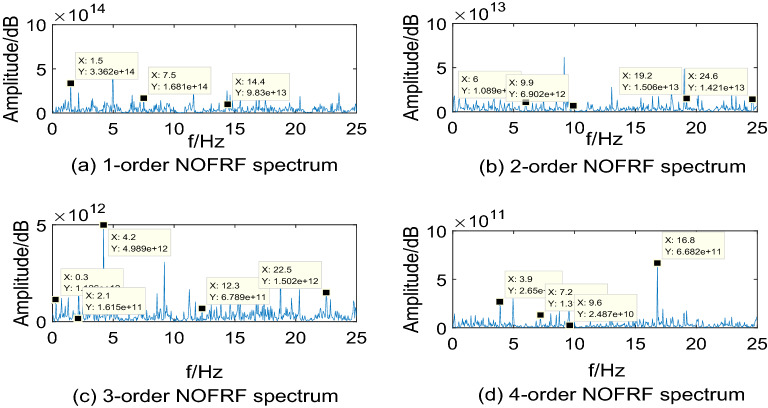
NOFRF spectrum of first 4-order in R3.

Making $i_{{d}} {(t) = A}_{{i}} {cos(0}{.6\pi t),(i = 1,2,3,4)}$ as input of system, where, ${\text{A}}_{{1}} {\text{ = 10,A}}_{{2}} {\text{ = 15,A}}_{{3}} {\text{ = 20,A}}_{{4}} { = 25}$. Then collecting the PMSM speed signal $\omega_{r}$ as output. The first 4-order NOFRF spectrums can be calculated by Eq. (7), which are shown as Figs. 5,6,7,8.

From Figs. 5,6,7,8, it can be seen that the difference of NOFRF spectrum between normal state and fault state is quite obvious, in other words, NOFRF spectrum is relatively sensitive to PMSM fault. Firstly, the key characteristics of first 4-order NOFRF spectrum in different states are quite different, such as, in the normal state, the key characteristics of first order NOFRF spectrum are 3 multiple-frequency, 5 multiple-frequency, and 52 multiple-frequency, and the key characteristics of fourth order NOFRF spectrum in normal state are mainly the odd multiple-frequency (3 multiple-frequency, 5 multiple-frequency, and 13 multiple-frequency),while the key characteristics of fourth order NOFRF spectrum in fault state caused by stator winding inter-turn short circuit are mainly even multiple-frequency (4-multiple-frequency, 24-multiple-frequency, 56-multiple-frequency, and 80-multiple-frequency).There are three types of multiple-frequency spectrums in normal state, and four types of multiple-frequency spectrum will be generated above two order NOFRF spectrum in fault state caused by composite reasons. The specific key characteristics distribution of NOFRF spectrum can be shown as Table 2.Secondly, even in the same state, the first 4-order NOFRF spectrums are different, with spectrum order increasing, the spectrum shows a trend of attenuation, the difference of spectrum can reach to three orders of magnitude. To some extent, the first 4-order NOFRF spectrums are the expression of fault information in four spatial dimensions, and the diversity of fault characteristics is enriched by the information difference in four dimensions. Therefore, it can effectively improve the accuracy of fault diagnosis by taking NOFRF spectrum as fault information for feature extraction and diagnosis.

**Table 2:** The key characteristics frequency points distribution of NOFRF spectrum in different states (basic frequency f = 0.3 HZ).

Label	1-order NOFRF	2-order NOFRF	3-order NOFRF	4-order NOFRF
R0	(3f, 2.4435e + 14)	(23f, 1.8720e + 13)	(3f, 9.6865e + 11)	(3f, 1.3881e + 11)
	(5f, 3.5140e + 14)	(38f, 1.2879e + 13)	(36f, 3.1755e + 12)	(5f, 1.3072e + 11)
	(52f, 1.7290e + 14)	(82f, 2.2777e + 13)	(41f, 7.4366e + 11)	(13f, 1.4454e + 11)
R1	(3f, 1.0296e + 14)	(2f, 6.6979e + 12)	(f, 9.9525e + 11)	(4f, 5.6902e + 10)
	(5f, 3.3486e + 14)	(13f, 1.2760e + 13)	(36f, 1.0542e + 12)	(5f, 1.2457e + 11)
	(23f, 1.3045e + 14)	(23f, 2.7025e + 13)	(41f, 7.5637e + 11)	(24f, 1.1361e + 11)
	None	(82f, 2.9755e + 13)	None	(56f, 2.8410e + 11)
	None	None	None	(80f, 1.2572e + 11)
R2	(3f, 1.4888e + 14)	(23f, 3.6127e + 13)	(3f, 5.9019e + 11)	(3f, 8.4576e + 10)
	(5f, 4.3188e + 14)	(38f, 1.7829e + 13)	(14f, 3.2507e + 12)	(5f, 1.6066e + 11)
	(48f, 1.4155e + 14)	None	(36f, 1.1508e + 12)	(32f, 5.0652e + 10)
	(52f, 1.7431e + 14)	None	None	(56f, 2.6736e + 11)
	(79f, 1.3929e + 14)	None	None	(64f, 1.7787e + 11)
R3	(5f, 3.3620e + 14)	(20f, 1.0886e + 13)	(f, 1.1265e + 12)	(13f, 2.6504e + 11)
	(25f, 1.6812e + 14)	(33f, 6.9016e + 12)	(7f, 1.6147e + 11)	(24f, 1.3173e + 11)
	(48f, 9.8300e + 13)	(64f, 1.5062e + 13)	(14f, 4.9892e + 12)	(32f, 2.4867e + 10)
	None	(82f, 1.4214e + 13)	(41f, 6.7887e + 11)	(56f, 6.6818e + 11)
	None	None	(75f, 1.5024e + 12)	None

Through the analysis above, we can draw conclusions as follows: (1) when PMSM power loss fault caused by stator winding inter-turn short circuit, the NOFRF spectrum of fourth order will appear even multiple-frequency spectrum. (2) when PMSM power loss fault caused by permanent magnetic leakage flux, the number of multiple-frequency point in NOFRF frequency of second order will decrease, and the fourth order NOFRF spectrum will appear more even multiple-frequency spectrums. (3) when PMSM power loss fault caused by stator winding inter-turn short circuit and permanent magnetic leakage flux together, the number of multiple-frequency point in NOFRF frequency of above two orders will increase, and the fourth order NOFRF spectrums have more even multiple-frequency spectrums.

## Experimental verification and discussions

**Figure 9:**
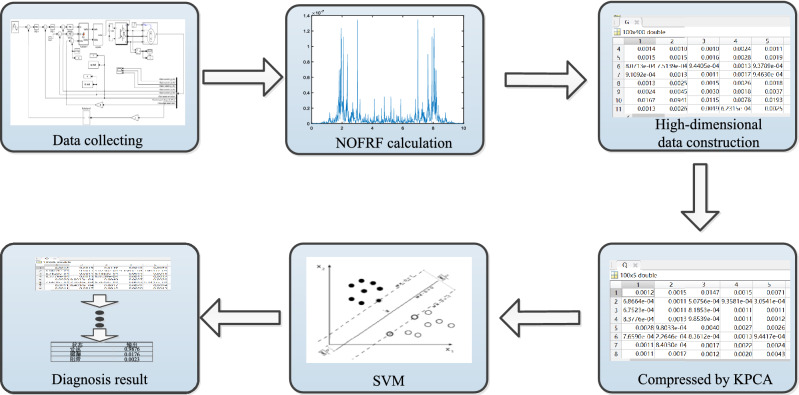
The process of experiment.

After the establishment of simulation model of industrial robot driving system, the system input and output are collected, the first four orders NOFRF spectrum of the system are obtained by the identification algorithm, and then the high-dimensional spectrum features are compressed and reduced by KPCA. Finally, the low-dimensional fault features in each state are put into SVM classifier for training and testing, and the fault identification and classification can be realized. The specific process can be shown in Fig. 9.

### NOFRF spectrum verification

**Figure10:**
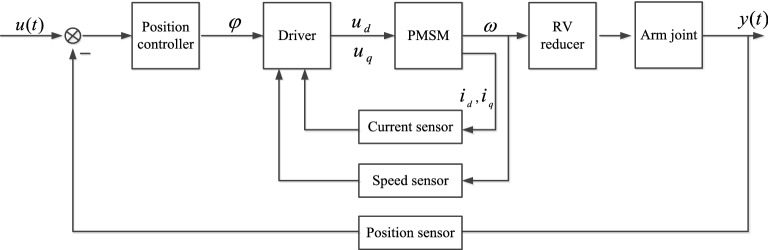
The structure of industrial robot system.

**Figure 11:**
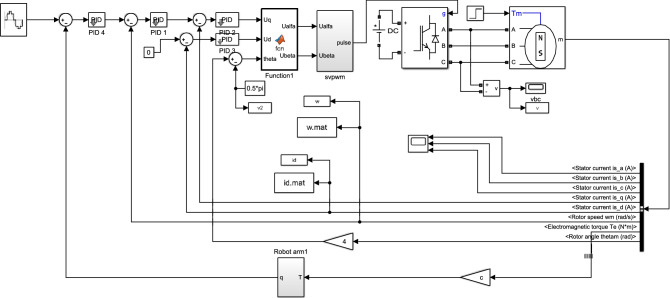
The simulation model of closed-loop drive system of robot.

In order to verify the effectiveness of NOFRF spectrum in real environment, this paper adopts the closed-loop system of industrial robot as shown in Fig. 10. The simulation model is established in Simulink, which is shown in Fig. 11. The input of robot system is selected as $u{(}t{)}\, = \,{10cos}\,({0}{.6}\pi t)$, the current sensor and speed sensor are adopted to collect the PMSM current signal $i_{d}$ and motor angular velocity speed $\omega_{r}$,respectively, then VSSLMS proposed in chapter 2.2 is adopted to calculate the first 4-order NOFRF spectrums.

**Figure 12:**
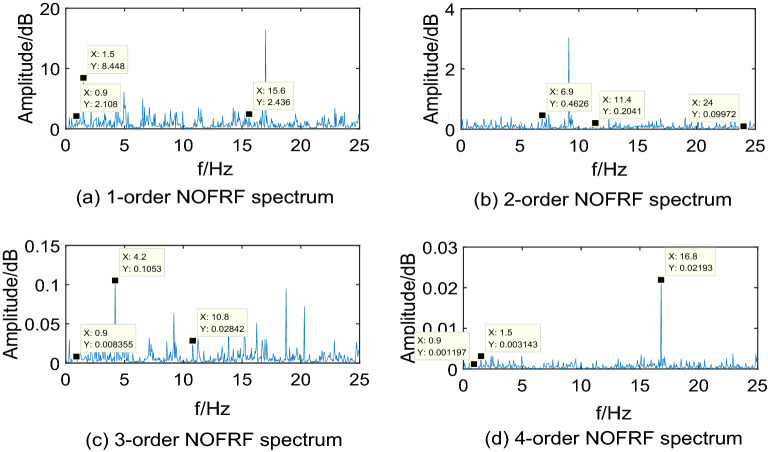
NOFRF spectrum of first 4-order in R0.

**Figure 13:**
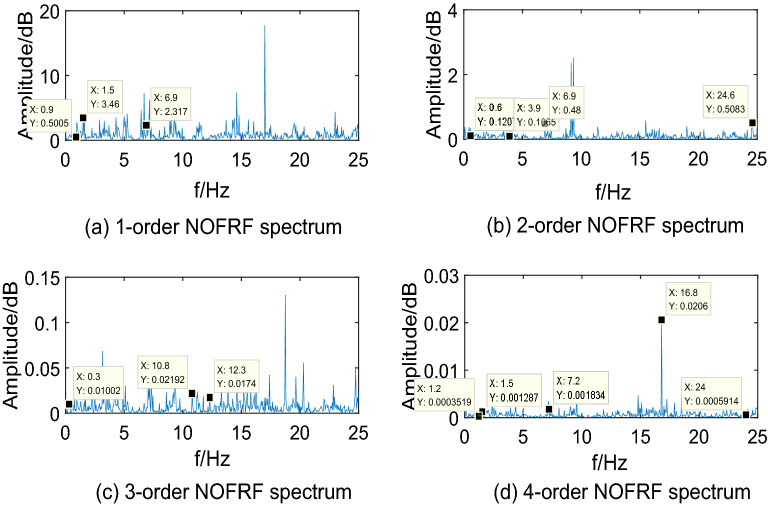
NOFRF spectrum of first 4-order in R1.

**Figure 14:**
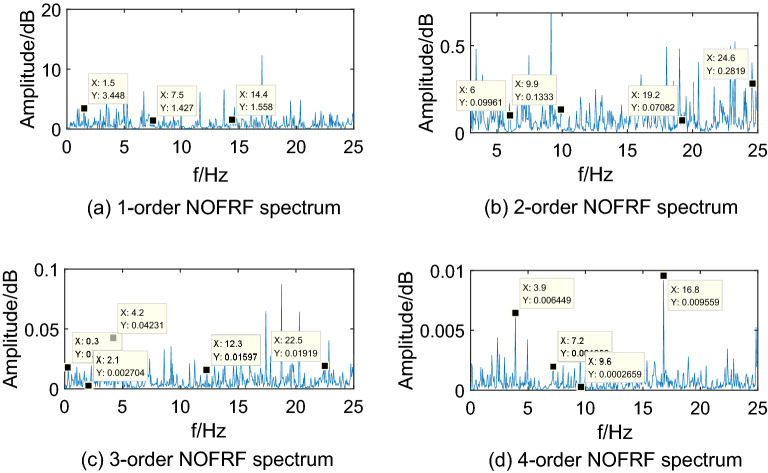
NOFRF spectrum of first 4-order in R2.

**Figure 15:**
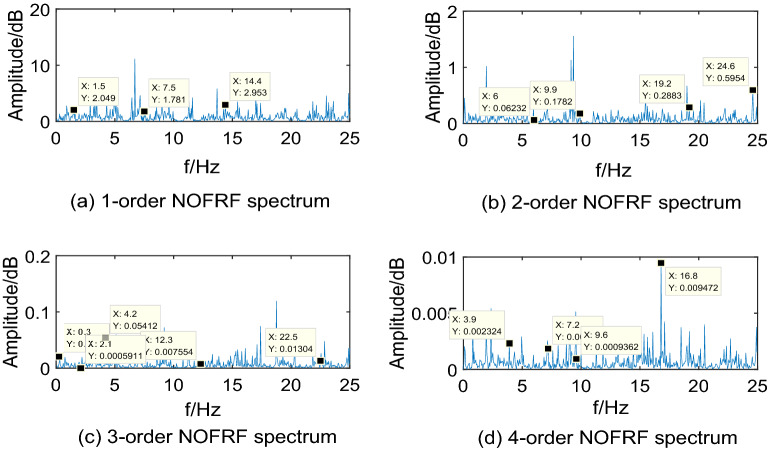
NOFRF spectrum of first 4-order in R3.

In the real environment, the first 4-order NOFRF spectrums of different states are obtained, which can be shown as Figs.12,13,14,15.

It can be seen from Figs.12,13,14,15 that the true value of first 4-order NOFRF spectrums obtained from data collecting by robot closed-loop system are similar to the theoretical value in terms of key characteristics distribution. For example, in the true value of fourth order NOFRF spectrum in fault state caused by stator winding inter-turn short circuit, it includes not only 5 multiple-frequency spectrum, but also four types of even fold frequency spectrums (4 multiple-frequency, 24 multiple-frequency, 56 multiple-frequency and 80 multiple-frequency), which is consistent with the key characteristics distribution of theoretical value. It is worthy to be noticed that the key characteristics of the theoretical value of third order NOFRF spectrum in normal state are 3 multiple-frequency, 36 multiple-frequency and 41 multiple-frequency, while the key characteristics of true value of third order NOFRF spectrum in this state are 3 multiple-frequency, 14 multiple-frequency and 36 multiple-frequency. The reason for this phenomenon is that there are some frequency points that do not converge in the process of NOFRF spectrum identification, which makes errors between the true value and the theoretical value.

### The verification of fault diagnosis effect

**Figure 16:**
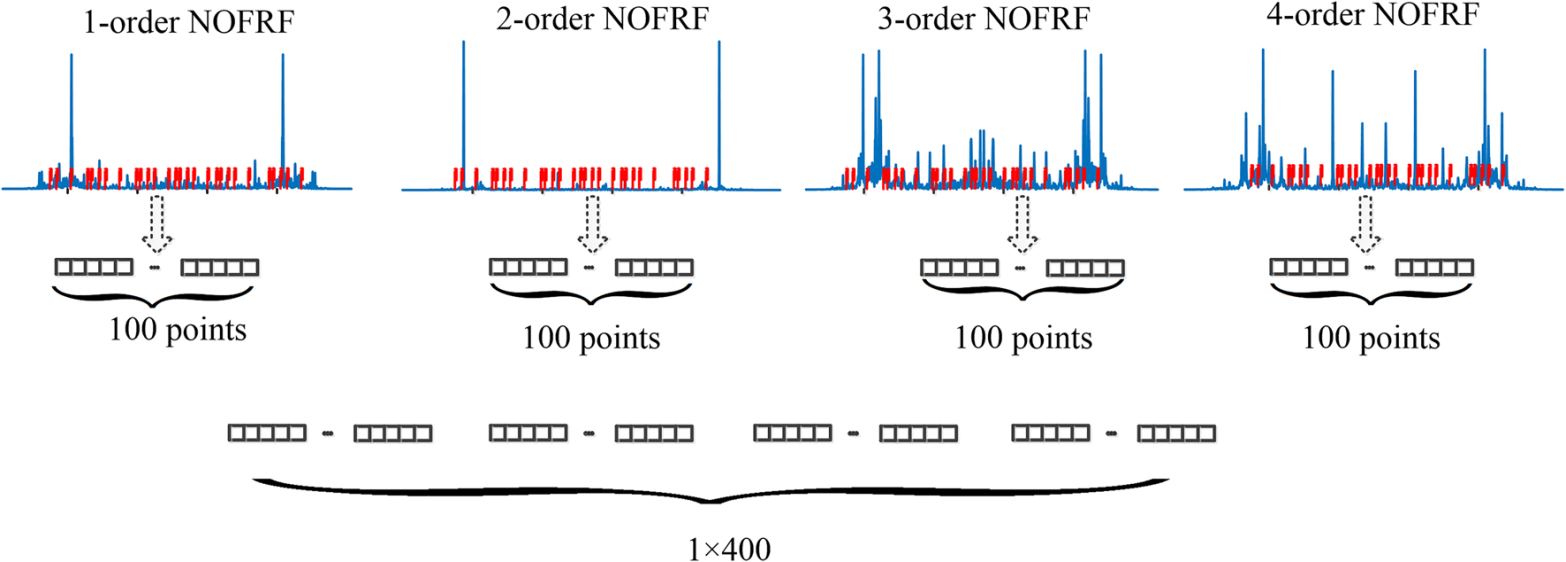
The NOFRF spectrum reorganize process.

In order to further verify the effectiveness of NOFRF spectrum as the fault information, the output signal *w*_*r*_ in time domain/frequency domain/time-frequency domain are adopted as fault information respectively for comparative experiment, and KPCA + SVM is adopted as classifier for fault diagnosis. In order to ensure the fairness of diagnosis experiment, the size of three forms of fault information in each state is 1 × 400. The time domain signal (TS) is generated by truncating using a window with the length of 400.The frequency domain information can be obtained by fast Fourier transform (FFT) of 1 × 400 time domain signal. The time-frequency domain information is obtained by short-time Fourier transform (STFT) of the time domain signal, after obtaining the 20 × 20 time-frequency map, tiling the time-frequency map to obtain 1 × 400 data. Each order NOFRF spectrum takes 100 points, and a total of 400 spectrum points can be obtained to form NOFRF spectrum of 1 × 400.The data reorganize process is shown as Fig. 16.

**Figure 17:**
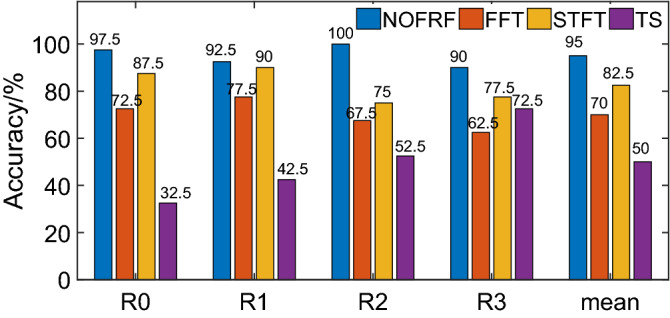
The accuracy of diagnosis with different fault information.

In the experiment, each state is repeated 200 times, which can be obtained the data set of 200 × 400. 80% of the data sets are randomly selected for training, and the rest are used for testing. The important parameters of KPCA and SVM are set as follows: In KPCA, Gaussian radial basis function is adopted as the kernel function, the width of kernel function is set to 170, and the cumulative contribution rate is set to 92%. As the classifier, SVM adopts the form of “one-versus-many”, in which the Gaussian radial basis functions are all selected as the kernel function, the parameters of the kernel function width are set to $10\sqrt 3$, the penalty factors are set to 0.4,and the training error is set to 10^−3^.The results of diagnosis with different fault information can be shown as Fig. 17.

**Figure 18:**
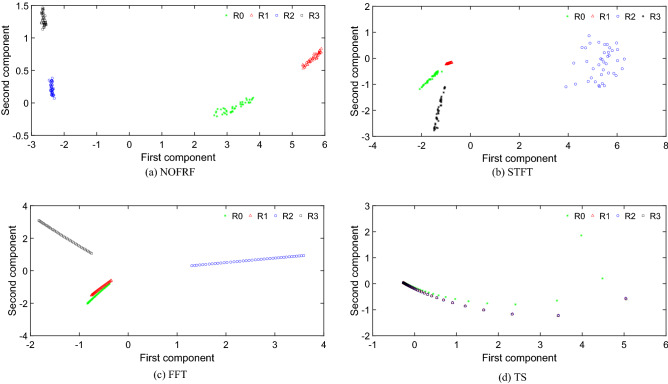
The visualization of different fault information.

As can be seen form Fig. 17, the accuracy of fault diagnosis by NOFRF, FFT, STFT and TS are 95%,70%,82.5% and 50%, respectively. It shows that compared with traditional fault information based on output signal (no matter what form it takes), NOFRF spectrum has strong advantages in fault characterization. The reason for such result is that NOFRF spectrum is a transfer spectrum, which can characterize the overall characteristics of the system and is very sensitive to the state changes. Meanwhile, NOFRF spectrum extracts the system fault information from four dimensions, which enriches the types of faults. While the three other types of fault information are based on output information, which represents the local information rather the global. It may not sensitive to the system state changes. What’s more, they may produce the redundancy of fault information, which is not conducive to the accurate judgment of classifier. In order to further illustrate the superiority of NOFRF spectrum for fault information characterization. The low dimensional features of four different fault information compressed by KPCA are visualized, which can be shown as Fig. 18.

From Fig. 18, it can be seen that after the NOFRF spectrum compressed by KPCA, the data characteristics of different states are more concentrated and that of same states are more divergent. Compared with NOFRF spectrum, after compression, although the STFT has obvious discrimination of data features in different states, the data features in R2 state are not aggregated, which easily cause the classifier to misjudge. After the fault information composed of FFT is compressed by KPCA, there is partial overlap between the data characteristics in R0 and R1 state. After the fault information composed of TS is compressed by KPCA, the data features of different states are completely mixed together, which may result in a high misjudgment rate by the classifier.

## Conclusions

In this paper, the fault mechanism of PMSM system is analyzed by NOFRF spectrum, and the multiple-frequency spectrum distribution rules of the first 4-order NOFRF spectrums under normal state and fault states caused by three reasons are obtained, the theoretical analysis results are verified in real system. Meanwhile, two types of fault characteristics analysis methods are compared. The main conclusions are as follows.

- (1) The NOFRF spectrum is more sensitive to PMSM faults and the NOFRF spectrums of different states vary greatly, which is manifested in form of high-frequency distribution. As fault characteristic, NOFRF spectrum can effectively reveal and distinguish the fault feature.
- (2) Compared with the existing diagnosis methods based on output signal in time domain/frequency domain/time-frequency domain, using the first 4-order NOFRF spectrums as fault information for the fault diagnosis of the robot drive system can achieve the highest accuracy.

The fault mechanism and diagnosis of industrial robot closed-loop drive system based on nonlinear spectrum is implemented. On one hand, it provides a new method for system fault information representation, on the other hand, it provides a theoretical basis for subsequent fault diagnosis research based on NOFRF spectrum. It’s a future work to adopt nonlinear spectrum to verify the effectiveness of fault diagnosis for other nonlinear systems.

## Data Availability

The data that support the finding of this study are available from the corresponding author upon reasonable request.
